# Exertional Rhabdomyolysis after an Extreme Conditioning Competition: A Case Report

**DOI:** 10.3390/sports6020040

**Published:** 2018-04-26

**Authors:** Ramires Alsamir Tibana, Nuno Manuel Frade de Sousa, Gabriel Veloso Cunha, Jonato Prestes, James W. Navalta, Fabricio Azevedo Voltarelli

**Affiliations:** 1 Department of Physical Education, Universidade Federal de Mato Grosso (UFMT), Cuiabá 78000-000, Brazil; faunesp8@yahoo.com.br; 2 Graduation Program on Physical Education, Catholic University of Brasilia, Brasilia 72000-000, Brazil; jonatop@gmail.com; 3 Laboratory of Exercise Physiology, Faculty Estacio of Vitoria, Vitoria 29000-000, Brazil; nunosfrade@gmail.com; 4 Undergraduate Program in Medicine, Catholic University of Brasilia, Brasilia 72000-000, Brazil; gabrielvelosoc@gmail.com; 5 Department of Kinesiology and Nutrition Sciences, University of Nevada, Las Vegas, NV 89154, USA; james.navalta@unlv.edu

**Keywords:** muscle damage, overreaching, extreme conditioning training, creatine kinase, rhabdomyolysis

## Abstract

This case report describes an instance of exercise-induced rhabdomyolysis caused by an extreme conditioning program (ECP) competition. A 35-year-old female presented with abdominal pain and soreness, which began one day after she completed two days of ECPcompetition composed of five workouts. Three days after competition, creatine kinase (CK) was 77,590 U/L accompanied by myalgia and abnormal liver function tests, while renal function was normal and this resulted in a diagnosis of rhabdomyolysis. A follow-up examination revealed that her serum level of CK was still elevated to 3034 U/L on day 10 and 1257 U/L on day 25 following the ECP competition. The subject reported myalgia even up to 25 days after the ECP competition. Exertional rhabdomyolysis can be observed in ECP athletes following competition and highlights a dangerous condition, which may be increasing in recent years due to the massive expansion of ECP popularity and a growing number of competitions. Future research should investigate the causes of rhabdomyolysis that occur as a result of ECP, especially training methods and/or tasks developed specifically for these competitions.

## 1. Introduction

Extreme conditioning programs (ECP) are a growing fitness regimen characterized by functional movements performed at high intensity and constantly varied movements [1,2]. Olympic weight lifting (snatch, clean and jerk), gymnastic movements (pull-ups, push-ups, handstand and sit-ups), and aerobic training (rowing, biking, and running) combined in an allotted time are typical within ECP workouts [3,4].

Recently, Tibana et al. [2] revealed that a single session of ECP (30 double-unders and 15 power snatches) induced a high immune (interleukin-6: 197 ± 109%; interleukin-10: 44 ± 52%) and metabolic (lactate: 1.20 ± 0.41 to 11.84 ± 1.34 mmol/L) response in trained subjects. In this sense, considering the high physical demand in this type of exercise program, adequate supervision of a professional is essential to avoid serious risks to health, especially syndromes such as rhabdomyolysis. Rhabdomyolysis is a syndrome characterized by muscle necrosis followed by the release of intracellular muscle contents into the circulation. Exertional rhabdomyolysis (ER) occurs in response to nonfamiliar and/or excessive, prolonged, or repetitive exercises, with eccentric characteristics [5]. Thus, due to the morbimortality associated with rhabdomyolysis, the best intervention would be the prevention [6]. On another hand, previous studies of ECP induced rhabdomyolysis demonstrated failures in training strategies for practitioners of the modality [7,8,9,10,11,12,13,14].

Few publications on exercise-induced rhabdomyolysis exist in the literature other than case reports. Recently, several case reports have been published, providing evidence that ECP-induced rhabdomyolysis is a growing issue (Table 1). However, previously case reports were in the traditional session of ECP. To the best of our knowledge, this is the first study case report of exercise-induced rhabdomyolysis following an extreme conditioning competition; this case occurred in a 35-year-old female athlete with several years of experience in ECP.

## 2. Case Report

A 35-year-old female (body weight 60.5 kg; height 1.55 m; body fat 16%; back squat 143 kg; front squat 125 kg; clean 97 kg; snatch 63 kg) without medical history of disease presented worsening abdominal pain approximately 24 h after completing a rigorous extreme conditioning competition (Table 2), which consisted of two days of five workouts. She was healthy overall and had been active in ECP over the previous five years and trained four or five times per week. The patient gave informed written consent for the use of her clinical and personal data in this paper.

The patient visited her physician one day after the ECP competition and was found to have a serum CK of 43,322 U/L. However, after receiving initial medical attention, she was sent home with instructions to take Tenoxicam (anti-inflammatory drug), bed rest, and drink plenty of water. On the third day post-competition, the pain and muscle swelling did not diminished, and she checked into an emergency room. At this stage her CK concentration was tested again and was 77,590 U/L. However, her kidney function, as indicated by blood urea, creatinine, sodium, and potassium concentrations was normal. On the other hand, her liver enzymes were elevated (aspartate aminotransferase (AST) of 477 U/L and alanine amino transferase (ALT) of 74 U/L). Chronological values of serum biochemistry and associated biomarkers over 25 days of follow-up are presented in Table 3. The patient was diagnosed with rhabdomyolysis by the medical attending physician and was treated with aggressive intravenous fluid resuscitation.

**Figure 1:**
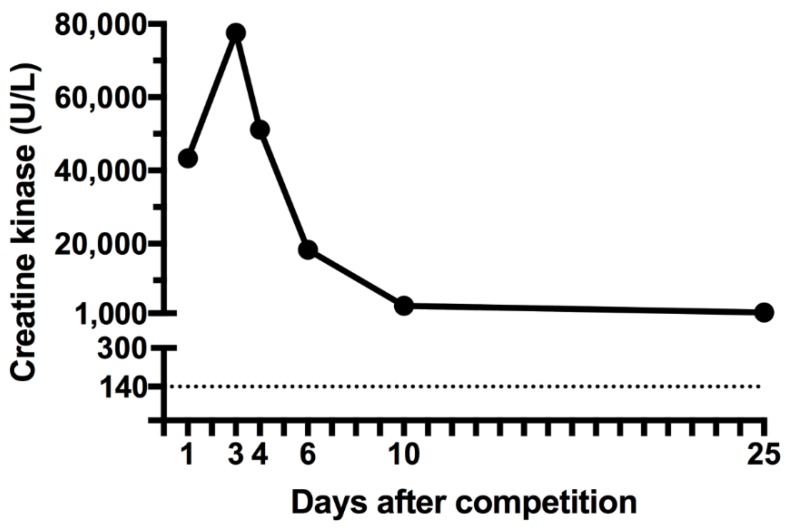
Creatine kinase concentration after 25 days of extreme conditioning program competition.

She was discharged on the fourth day of hospitalization and she was advised to avoid intense exercise. A follow-up examination revealed that her serum level of CK was still elevated to 3034 U/L on the 10th day and 1257 U/L on the 25th day following the ECP competition (Figure 1). The subject reported myalgia even 25 days after the ECP competition.

## 3. Discussion

The subject of this case report was a 35-year-old female accustomed to regular ECP and exhibited rhabdomyolysis induced for an unaccustomed exercise (GHD). However, she was diagnosed with rhabdomyolysis after completing an EC competition lasting two days and composed of five workouts. Rhabdomyolysis is defined by a rise in CK to five times the upper limit of normal values, ranging from 1500 to over 100,000 U/L [15,16] and the symptoms associated with rhabdomyolysis include myalgia, muscle weakness, myoglobinuria, and dark urine [15].

**Figure 2:**
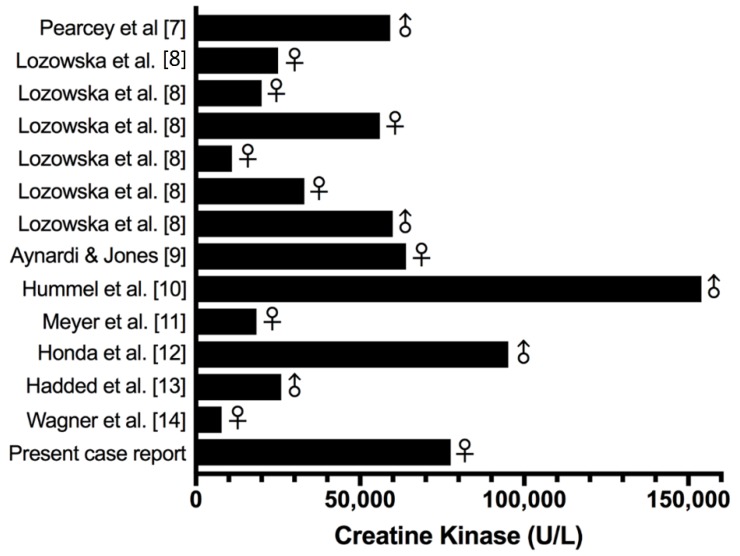
Creatine kinase concentration of case reports presented in the literature and the present case report.

In the current case, one day after the competition, the female athlete presented with severe myalgia and CK higher than the upper limit of normal values (43,322 U/L). Only at the third day after the competition, when CK was 77,590 U/L, myoglobin was 1350 ng/mL and severe myalgia continued to present, was the diagnosis of rhabdomyolysis was made, even with normal renal function. Other cases of exertional rhabdomyolysis due to ECP have reported myalgia with CK concentrations higher than 10,000 U/L [7,8,9,10,11,12,13] and only one with CK activity lower than 10,000 U/L [14] (Figure 2). Thus, the initial elevated CK concentration of more than 10,000 U/L after ECP training or competition could have been sufficient for medical professionals to recommend further testing at an emergency care hospital. Although rhabdomyolysis is more common in untrained subjects, in the present case report and in six subjects of the case reports published in the literature, rhabdomyolysis occurred in physically active and well-trained subjects, who regularly participated in ECP.

Among the potential complications associated with rhabdomyolysis, acute renal failure, compartment syndrome, and disseminated intravascular coagulation can be life threatening [15,16]. Acute renal injury is estimated to occur in approximately one-third of rhabdomyolysis patients [15,16]. Direct injury to the kidneys by the accumulation of myoglobin is the main cause of the acute renal failure associated with rhabdomyolysis [15,17]. It is reasonable to conclude that higher serum levels of CK, such as >5000 U/L, could be associated with the development of acute renal failure [15]. When acute renal failure is suspected in rhabdomyolysis patients, aggressive fluid replacement as well as dialysis should immediately be considered [15,16,18]. In our case report, no serious complications occurred, while there was a marked elevation in the patient’s serum level of CK and myoglobin. The rate of acute renal failure among exertional rhabdomyolysis patients has been reported to be lower than that among non-exertional rhabdomyolysis patients [19]. Another early complication if rhabdomyolysis is untreated is electrolyte and fluid abnormalities, with later severe consequences including cardiac dysrhythmias and hypercalcemia [20]. Another interesting point is whether highly trained subjects may support higher levels of CK without severe renal complications.

The circumstances of this case report are somewhat similar to other cases involving ECP performed with a high number of repetitions of an exercise at high intensity. In comparison, this case report may be more relevant because it occurred during an EC-competition in a well-trained woman who had participated in ECP for years. In this sense, coaches and trainers of ECP should be aware of the risk and may consider prescribing lower volume and intensity sessions in non-usual exercises weeks before a competition to minimize the risk of rhabdomyolysis. The inclusion of unaccustomed exercise with volume and intensity similar to a competition is to induce a cellular protection, this phenomenon is known as the ‘repeated bout effect’ [21].

Finally, we must rethink current ECP strategies to improve athletic performance because, unfortunately, exertional rhabdomyolysis is often becoming an outcome for ECP practitioners. We believe that the periodization of the program considering the progressive increase in volume and intensity in non-usual exercises could be the best way to prevent some undesired events such as rhabdomyolysis. Exertional rhabdomyolysis can be seen in ECP athletes following competition. This case report highlights ECP-induced rhabdomyolysis, a condition which may be increasing in recent years due to the massive expansion of ECP popularity and a growing number of competitions. Future research should investigate the causes of rhabdomyolysis during ECP, especially training methods and/or developed tasks during the competitions.

## Figures and Tables

**Table 1:** Case reports showing extreme conditioning program-induced rhabdomyolysis.

Authors	Subject	Physical Status	Protocol of ECP
Pearcey et al. [7]	Man	Athlete who was acutely trained (approximately 3 months).	48 alternating sets (60 s duration) of push-up and pull-up variations. The subject performed the maximum number of repetitions possible of push-ups or pull-ups in each set. The total exercise duration was 48 min. The subject performed approximately 400 push-ups and approximately 200 pull-ups in 48 min.
Lozowska et al. [8]	Five of 6 patients were women	Three of the 6 patients were very physically fit before experiencing rhabdomyolysis, having participated in CrossFit for months to years. The remaining 3 patients were less fit and sustained rhabdomyolysis after their first encounter with CrossFit.	Non-informed.
Aynardi & Jones [9]	A 43-year old, African American female	She was healthy overall and had been active in multiple gym-related exercise programs over the past 10 years.	The ECP consisted of a standard warm-up followed by 3 sets of chin-ups that were performed until “failure” lasting approximately 20 min.
Hummel et al. [10]	A 15-year-old previously healthy male	Non-informed.	Intense CrossFit^®^ workout.
Meyer et al. [11]	A previously healthy 31-year-old woman	She was exercising regularly 4 times per week, performing pushups, running, and other physical workouts.	The subject denied recent trauma or illness, but reported performing a variety of high intensity exercises such as pushups, plyometrics, and weightlifting at CrossFit.
Honda et al. [12]	A previously healthy 37-year-old man	He had exercised regularly but had never performed such intense training before.	Intense exercise training that included 100 pushups, 100 exercises using a 20-kg dumbbell, 50 lifts using a 10-kg weight.
Hadded et al. [13]	Man	He reports having had 5 previous days of exercise but did not involve CrossFit type training.	Non-informed.
Wagner et al. [14]	Woman	A healthy 21-year-old Caucasian female was participating in an organized, extreme exercise workout session conducted at a fitness center.	The exercise session consisting of performing a designated number of pushups in one minute. The protocol dictated 5 pushups in the first minute, 10 in the second, and adding 5 pushups each minute until participants can no longer continue. She recalls completing 6 rounds of increasing repetitions in each minute, thereby performing 105 pushups in 6 min.

**Table 2:** Schematic representation of the sessions during extreme conditioning program competition lasting two days.

Schedule	Day 1	Day 2
Workout 1 2:00 p.m.	21 chest-to-bar pull-ups 21 thrusters (40 kg) 9 chest-to-bar pull-ups 9 thrusters (40 kg)	-
Workout 2 05:00 p.m.	60 GHD sit ups (unaccustomed exercise) 15 toes-to-bar	-
Workout 3 1:00 p.m.	-	1 RM of squat snatch + overhead squat
Workout 4 04:00 p.m.	-	AMRAP during 5 min of strict handstand push-ups
Workout 5 5:30 p.m.	-	40 deadlifts (45 kg) 20 kettlebells clean and jerks (24 kg) 5 bar muscle-ups

GHD, glutes-hamstring developer; RM, repetition maximum; AMRAP, as many rounds as possible.

**Table 3:** Chronological values of serum biochemistry and associated biomarkers.

Biomarkers	Reference Values	1st Day Post-ECC	3rd Day Post-ECC	4th Day Post-ECC	6th Day Post-ECC	6th Day Post-ECC	10th Day Post-ECC	25th Day Post-ECC
Creatine kinase (CK) (U/L)	26–140	43.322	77590	51.195	18.347	18.287	3034	1257
Lactic dehydrogenase (LDH) (U/L)	240–248	-	-	1.835	555	607	-	-
Myoglobin (ng/mL)	10–46	-	1350	-	-	-	24	58
C-reactive protein (CRP) (mg/dL)	-	0.46	1.18	0.84	0.53	0.55	-	-
Erythrocyte sedimentation rate (ESR) (mm/h)	<10	27	14	22	9	8	-	-
Urea (mg/dl)	10–50	26	17	14	13	13	-	-
Creatinine (mg/dL)	0.60–1.10	1.22	0.99	0.90	1.02	1.01	-	-
Potassium (mEq/L)	3.5–4.5	4.3	4.6	3.9	4.2	4.3	-	-
Sodium (mEq/L)	136–145	139	142	139	139	139	-	-
Calcium (mg/dL)	8.6–10.3	9.7	9.3	-	8.2	8.4	-	-
Chlorine (mEq/L)	98–107	105	-	108	-	109	-	-
Phosphorus (mg/dL)	2.5–4.5	4.9	-	2.7	-	3.8	-	-
Magnesium (mg/dL)	1.6–2.6	2.1	-	1.9	-	1.7	-	-
Aspartate transaminase (AST) (U/L)	<32	17	477	969	682	322	108	37
Alanine transaminase (ALT) (U/L)	<33	12	74	208	159	100	78	20
Gamma-glutamyl transferase (GGT) (U/L)	8–41	-	9	7	5	9	-	-
Alkaline phosphatase (FA) (U/L)	35–104	-	55	54	49	44	-	-
Lactate (mg/dL)	5.7–22	-	16.3	14.3	23.7	10.5	-	-
Leukocytes (cells/mm^3^)	3.500–10.500	10.860	8.240	6.960	7.220	6.820	11220	8480
